# Bing–Neel Syndrome and Coexisting Pituitary Macroadenoma in a Patient with Waldenström Macroglobulinemia Revealed by ^18^F-FDG and ^68^Ga-Pentixafor PET/CT

**DOI:** 10.3390/diagnostics13071334

**Published:** 2023-04-03

**Authors:** Qingqing Pan, Yaping Luo, Xinxin Cao, Jian Li, Jun Feng

**Affiliations:** 1 Department of Nuclear Medicine, Chinese Academy of Medical Sciences and Peking Union Medical College Hospital, Beijing 100730, China; 2 Beijing Key Laboratory of Molecular Targeted Diagnosis and Therapy in Nuclear Medicine, Beijing 100730, China; 3 Department of Hematology, Chinese Academy of Medical Sciences and Peking Union Medical College Hospital, Beijing 100730, China

**Keywords:** Waldenström’s macroglobulinemia, Bing–Neel syndrome, ^68^Ga-Pentixafor, FDG, PET/CT

## Abstract

A 63-year-old man presenting with peripheral neuropathies was diagnosed of Waldenström’s macroglobulinemia, and Bing–Neel syndrome was subsequently confirmed via cerebrospinal fluid examinations. Besides involvement in bone marrow, lymph nodes, as well as the thoracic and sacral nerve root,^ 68^Ga-Pentixafor PET/CT detected active tracer uptake in bilateral choroid plexus, which was negative in ^18^F-FDG PET/CT, possibly suggesting the involvement of Bing–Neel syndrome. The coexisting pituitary macroadenoma was FDG-avid but negative in ^68^Ga-Pentixafor PET/CT. After six cycles of chemotherapy, the follow-up PET/CT showed complete remission of the previous disease, including the high uptake of ^68^Ga-Pentixafor in choroid plexus. However, the hypermetabolic pituitary macroadenoma remained unchanged.

**Figure 1:**
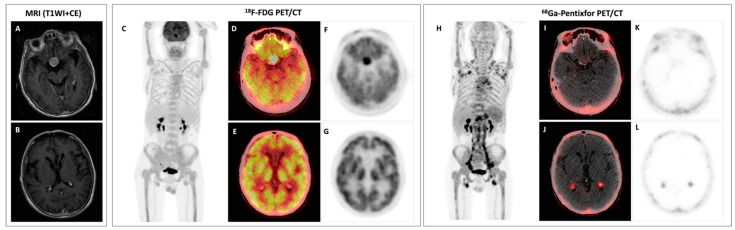
Baseline evaluation, axial contrast-enhanced T1-weighted MRI (**A**,**B**), MIP image of the ^18^F-FDG PET (**C**), axial fusion ^18^F-FDG PET/CT (**D**,**E**), axial ^18^F-FDG PET (**F**,**G**), MIP image of the ^68^Ga-Pentixafor PET (**H**), axial fusion ^68^Ga-Pentixafor PET/CT (**I**,**J**), and axial ^68^Ga-Pentixafor PET (**K**,**L**). This patient (male, 63 years old) had a numbness and amyosthenia in the right foot 2 months prior, and his symptom progressively developed to the bilateral lower and upper extremities. Electromyography showed demyelination and axonal injury of sensorimotor peripheral nerves in the lower extremities. Laboratory tests showed anemia (hemoglobin 94 g/L) and elevated serum IgM (30.49 g/L; reference: 0.4–2.3 g/L). Serum protein and immunofixation electrophoresis were positive for monoclonal protein (18.5 g/L, IgM-κ). Bone marrow biopsy showed infiltration with lymphoplasmacytoid cells accounting for 10% of the cells in bone marrow. Therefore, the diagnosis of Waldenström’s macroglobulinemia (WM), a low-grade lymphoma, was established. Since the patient had a chief complaint of peripheral neuropathies, Bing–Neel syndrome (central nervous system involvement in WM) was considered, as other common causes of peripheral neuropathies in WM—paraprotein-related peripheral neuropathies, cryoglobulinemia, light chain amyloidosis—were excluded by sural nerve biopsy and negative serum cryoglobulin. The cerebrospinal fluid examinations revealed an M-spike of 0.19 g/L (IgM-κ restricted) and positive mutation of MYD88^L265P^ (an important marker for diagnosing WM [1,2]), supporting the diagnosis of Bing–Neel syndrome. The patient then underwent a brain MRI, which was unremarkable except for a pituitary macroadenoma (**A**). In ^18^F-FDG PET/CT, the pituitary macroadenoma was hypermetabolic ((**D**,**F**), SUVmax 10.4); additionally, diffusely and homogenously increased uptake in bone marrow and involvement in lymph nodes were also depicted (**C**). The patient then underwent ^68^Ga-Pentixafor PET/CT, a chemokine receptor CXCR4-targeted imaging, which we previously reported as being superior to ^18^F-FDG in patients with WM [3]. MIP image of the ^68^Ga-Pentixafor PET showed markedly increased radioactivity throughout the axial and appendicular skeleton, as well as multiple lymph nodes in the neck, axilla, para-aortic, pelvic, and inguinal areas with increased uptake (**H**).Surprisingly, ^68^Ga-Pentixafor PET/CT showed active tracer uptake in bilateral choroid plexus ((**J**,**L**), SUVmax 4.0) that was negative with ^18^F-FDG (**E**,**G**) and also with normal signal intensity and contrast-enhancement in MRI (**B**); the FDG-avid pituitary macroadenoma was negative with ^68^Ga-Pentixafor (**I**,**K**). Furthermore, involvements in the thoracic nerve root and sacral nerve root were also detected.

**Figure 2:**
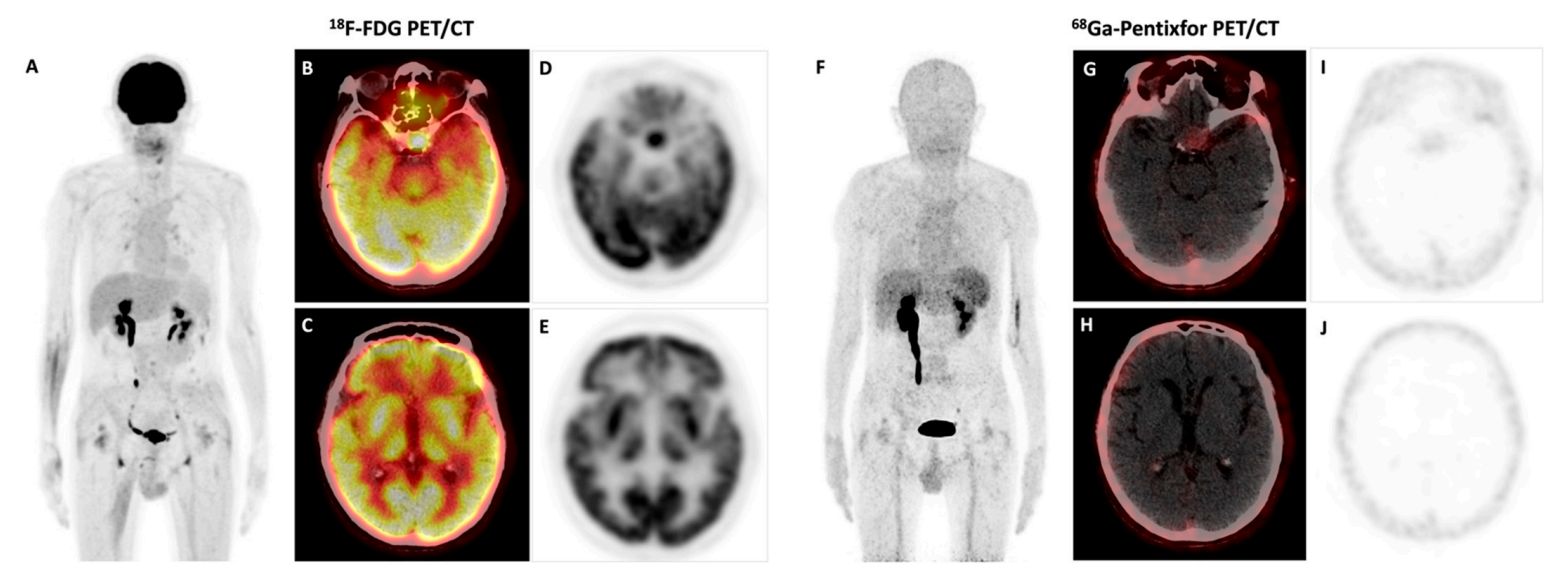
After six cycles of chemotherapy, the patient underwent follow-up PET/CT with both ^18^F-FDG and ^68^Ga-Pentixafor. MIP image of the ^18^F-FDG PET (**A**), axial fusion ^18^F-FDG PET/CT (**B**,**C**), axial ^18^F-FDG PET (**D**,**E**), MIP image of the ^68^Ga-Pentixafor PET (**F**), axial fusion ^68^Ga-Pentixafor PET/CT (**G**,**H**), and axial ^68^Ga-Pentixafor PET (**I**,**J**). The previous high uptake of ^68^Ga-Pentixafor in the choroid plexus returned to normal. There was also a complete remission of bone marrow, lymph node, and nerve roots involvement in both ^68^Ga-Pentixafor and ^18^F-FDG PET/CT. However, the manifestations of the pituitary macroadenoma remained unchanged (SUVmax 11.2 in ^18^F-FDG PET/CT). Bing–Neel syndrome is a rare complication involving almost 1% of WM patients [4]. About one-third of patients with Bing–Neel syndrome had symptoms of peripheral neuropathies [4,5]. Leptomeningeal infiltration was most commonly seen, followed by parenchymal and dural involvement [4,6]. Involvement in the choroid plexus is rare. MRI can be used to detect central nervous system involvement in Bing–Neel syndrome, but it lacks specificity. In a previous case report of Bing–Neel syndrome, the lesions shown in MRI were hypometabolic in ^18^F-FDG PET/CT [7], which was consistent with our case. Two other cases reported FDG-avid central nervous system involvement of Bing–Neel syndrome [8,9]. ^18^F-FDG showed pronounced physiological accumulations in the brain, which may omit the pathological findings. However, the physiologic uptake of ^68^Ga-Pentixafor is very low in the head, resulting in excellent tumor-to-background contrast and a higher detection rate of cerebral lesions. Thus, as there is a high level of CXCR4 expression in B-cells of WM [1,10], ^68^Ga-Pentixafor should be further assessed as a potential tracer for the evaluation of WM, including the involvement of the central nervous system.

## Data Availability

The data presented in this study are available on request from the corresponding author.

## References

[1] Hunter Z.R., Yang G., Xu L., Liu X., Castillo J.J., Treon S.P. (2017). Genomics, Signaling, and Treatment of Waldenstrom Macroglobulinemia. J. Clin. Oncol..

[2] Mazzucchelli M., Frustaci A.M., Deodato M., Cairoli R., Tedeschi A. (2018). Waldenstrom’s Macroglobulinemia: An Update. Mediterr. J. Hematol. Infect. Dis..

[3] Luo Y., Cao X., Pan Q., Li J., Feng J., Li F. (2019). ^68^Ga-Pentixafor PET/CT for Imaging of Chemokine Receptor 4 Expression in Waldenström Macroglobulinemia/Lymphoplasmacytic Lymphoma: Comparison to ^18^F-FDG PET/CT. J. Nucl. Med..

[4] Minnema M.C., Kimby E., D’Sa S., Fornecker L.-M., Poulain S., Snijders T.J., Kastritis E., Kremer S., Fitsiori A., Simon L. (2017). Guideline for the diagnosis, treatment and response criteria for Bing-Neel syndrome. Haematologica.

[5] Castillo J.J., D’Sa S., Lunn M., Minnema M.C., Tedeschi A., Lansigan F., Palomba M.L., Varettoni M., Garcia-Sanz R., Nayak L. (2016). Central nervous system involvement by Waldenstrom macroglobulinaemia (Bing-Neel syndrome): A multi-institutional retrospective study. Br. J. Haematol..

[6] Fitsiori A., Fornecker L.M., Simon L., Karentzos A., Galanaud D., Outteryck O., Vermersch O., Pruvo P., Gerardin J.-P., Lebrun-Frenay E. (2019). Imaging spectrum of Bing-Neel syndrome: How can a radiologist recognise this rare neurological complication of Waldenstrom’s macroglobulinemia?. Eur. Radiol..

[7] Bund C., Lhermitte B., De Seze J., Kremer S., Namer I.J. (2018). FDG PET and MRI Findings in a Case of Bing-Neel Syndrome. Clin. Nucl. Med..

[8] Illarramendi O.A., Flynt L., Wong F. (2019). 18F-FDG PET/CT in the Evaluation of Bing-Neel Syndrome. J. Nucl. Med. Technol..

[9] Riaz S., Priftakis D., Arulogun S., Wan S., Bomanji J. (2020). Before and After Treatment Characterization of Cerebrospinal Disease in Bing-Neel Syndrome Using 18F FDG PET/MRI. Clin. Nucl. Med..

[10] Ngo H.T., Leleu X., Lee J., Jia X., Melhem M., Runnels J., Moreau J., Burwick A.-S., Azab N., Roccaro A.K. (2008). SDF-1/CXCR4 and VLA-4 interaction regulates homing in Waldenstrom macroglobulinemia. Blood.

